# A Mitochondrion-Targeting Protein (B2) Primes ROS/Nrf2-Mediated Stress Signals, Triggering Apoptosis and Necroptosis in Lung Cancer

**DOI:** 10.3390/biomedicines11010186

**Published:** 2023-01-11

**Authors:** Hsuan-Wen Chiu, Shao-Wen Hung, Ching-Feng Chiu, Jiann-Ruey Hong

**Affiliations:** 1 Laboratory of Molecular Virology and Biotechnology, Institute of Biotechnology, National Cheng Kung University, Tainan 701, Taiwan; 2 Department of Biotechnology and Bioindustry, National Cheng Kung University, Tainan 701, Taiwan; 3 Division of Animal Industry, Animal Technology Research Center, Agricultural Technology Research Institute, Hsinchu 300, Taiwan; 4 Graduate Institute of Metabolism and Obesity Sciences, College of Nutrition, Taipei Medical University, Taipei 11031, Taiwan; 5 Graduate TMU Research Center of Cancer Translational Medicine, Taipei Medical University, Taipei 11031, Taiwan

**Keywords:** anticancer peptide, lung cancer cell, apoptosis, necroptosis, mitochondrial targeting, NOD/SCID mice, oxidative stress

## Abstract

The betanodavirus B2 protein targets mitochondria and triggers mitochondrion-mediated cell death signaling in lung cancer cells; however, its molecular mechanism remains unknown. In this study, we observed that B2 triggers hydrogen peroxide/Nrf2-involved stress signals in the dynamic regulation of non-small lung cancer cell (NSCLC)-programmed cell death. Here, the B2 protein works as a necrotic inducer that triggers lung cancer death via p53 upregulation and RIP3 expression, suggesting a new perspective on lung cancer therapy. We employed the B2 protein to target A549 lung cancer cells and solid tumors in NOD/SCID mice. Tumors were collected and processed for the hematoxylin and eosin staining of tissue and cell sections, and their sera were used for blood biochemistry analysis. We observed that B2 killed an A549 cell-induced solid tumor in NOD/SCID mice; however, the mutant ΔB2 did not. In NOD/SCID mice, B2 (but not ΔB2) induced both p53/Bax-mediated apoptosis and RIPK3-mediated necroptosis. Finally, immunochemistry analysis showed hydrogen peroxide /p38/Nrf2 stress strongly inhibited the production of tumor markers CD133, Thy1, and napsin, which correlate with migration and invasion in cancer cells. This B2-triggered, ROS/Nrf2-mediated stress signal triggered multiple signals via pathways that killed A549 lung cancer tumor cells in vivo. Our results provide novel insight into lung cancer management and drug therapy.

## 1. Introduction

The aquatic betanodavirus contains two RNA genomes, i.e., segment one (RNA1) and segment two (RNA2), which are 3.1 and 1.4 kb in length, respectively. These segments can individually cause brain damage in fish [1,2,3,4,5,6]. Using genomic RNA replication, the RNA1 of aquatic betanodavirus synthesizes a subgenomic RNA3 segments in an early replication stage, which encodes two non-structural proteins named B1 and B2 [1,7,8]. In the RGNNV strain, B1 has been observed to exhibit anti-cell death activity [9], which may affect viral replication. Recently, B2 has been shown to play a dual function as either a suppressor, blocking host siRNA silencing in alpha- [10,11] and betanodaviruses [7], or as a kill gene that directly targets the mitochondria and inhibits mitochondrial complex II activity, blocking ATP synthesis in vitro and in vivo [12]. Furthermore, B2 triggers cancer cell death, such as that of lung adenocarcinoma cells and breast adenocarcinoma cells [13]. However, all these studies have failed to uncover the molecular mechanism for this B2 activity.

In normal cells, reactive oxygen species (ROS) are continuously regulated using intricate feedback loops that accentuate their importance in biochemical metabolism processes. ROS are also influence dynamic tumor microenvironments, initiating cancer metastasis, survival, and angiogenesis under different conditions [14]. For instance, ROS can activate survival signaling pathways in cancer cells, including the MAPK/ERK1/2, phosphoinositide-3-kinase/protein kinase B (PI3K/Akt), p38, and c-Jun N-terminal kinase (JNK) pathways. These, in turn, lead to transcriptional changes in downstream genes, such as vascular endothelial growth factor (VEGF) and matrix metalloproteinases (MMPs). However, at higher concentrations, ROS shift to signal apoptosis in cancerous cells. Thus, the choice between tumorigenesis and apoptosis depends upon the ROS concentrations within the cells [15]. Additionally, ROS generation levels and cellular antioxidant enzyme systems can both be modulated in malignantly transformed cells. This is achieved by using different transcriptional factors, such as NF-κB and Nrf2. ROS-mediated signaling cascades are also regulated by other key factors, such as inflammatory responses, apoptosis inhibition, induction of tumor proliferation, and the triggering of metastasis [16,17,18].

Apoptosis is usually induced as a natural part of aging and development, working as a common mechanism that maintains appropriate cell population levels within organisms. Cell death signaling can be performed simultaneously, as a defensive action triggered by innate or adaptive immune responses during foreign pathogen infection. At present, there are two commonly studied apoptotic pathways: one is the intrinsic pathway, which presents a mitochondrion-mediated death signal, and the other is the extrinsic pathway performed by receptor-mediated signals, such as Fas or TNF [19,20]. The latter is well-reported and involves proapoptotic transmembrane death receptors, the ligation of which ultimately leads to the execution of extrinsic apoptosis through the activation of caspase-8. However, this protein can also be activated by other receptors that act in response to viral DNA, such as RIG-I-like and Toll-like receptors [21,22]. The intrinsic pathway is related to pathways of irreparable genomic damage, activation of oncogenes, growth factor withdrawal, checkpoint violations, hypoxia, and other metabolic stress or internal trauma [23].

Necrosis is suggested to be a series of programmed events, collectively termed necroptosis, rather than a series of unregulated processes [24]. Some receptor families, such as FasL, TRAIL, and TNFα, can use ligands that induce apoptosis and trigger a switch to necroptosis. In addition to these ligands, receptor-interacting protein (RIP) kinases also greatly participate in the regulation of cell death and survival processes [21]. The RIP family has seven proteins, each with a kinase domain (KD) that further modulates function. RIPK3 has been reported to regulate the necroptotic pathway, which is an activity that is of particular interest in the present study [22,25].

Recently, functional peptides have gradually been developed as emerging therapies to treat a variety of illnesses, such as with anticancer peptides (ACPs) [26]. Some of the advantages of these therapeutic peptides include tissue specificity and low cytotoxicity. Anticancer effects may occur as a direct consequence of a peptide binding to its target or by conjugating the peptide to a chemotherapeutic drug or radionuclide that targets cancer cells [27]. At the cell surface, ACPs target proteins that allow them to commence the internalization process, successfully crossing the cell membrane [26,27,28]. ACPs induce apoptosis via various mechanisms, including cell death pathways, DNA repair pathways, cell cycle regulation, disruption of cell signaling pathways, immune regulation, tumor angiogenesis inhibition, membrane disruption, and subsequent necrosis [26,27,28]. In our system, B2 derived from a viral gene was able to trigger an ROS/Nrf2-mediated stress response, activating multiple signals and eventually leading to induction of host cell death.

Our group previously used in vitro and in vivo systems (fish cells and zebra fish models) to study the effect of B2 protein on cell death induced by ATP depletion [8,12,13,29]. However, such studies have not elucidated the function of the B2 protein in mitochondrion-mediated cell death triggering. Here, we used the novel viral B2 protein to target solid tumors and A549 lung cancer cells. Such experiments are used to study ROS/Nrf2-mediated stress signaling, which is known to induce cell death. The betanodavirus B2 protein, as a necrotic inducer, hints at a novel perspective on lung cancer therapy.

## 2. Materials and Methods

### 2.1. Cancer Cell Culture

A549 (ATCC, CCL-185™), a human lung cancer cell line, was cultured in a specific medium comprising 2 mM L-glutamine, 100 mg/mL of streptomycin, 100 U/mL of penicillin (Invitrogen), and 10% fetal bovine serum (Gibco). Cells were seeded in 10 cm^2^ Petri dishes or 6-well culture plates within incubators set to 5% CO_2_ and 37 °C [12].

### 2.2. Plasmid Constructions

Two versions of the RGNNV B2 gene included either a novel targeting sequence (41RTFVISAHAA50) or a mutated sequence as a mutant form and were cloned further into pEYFP-C1 (Clontech), p3XFLAG-*myc*-CMV-26 (Sigma), and pcDNA3.1 (Clontech Laboratories, Palo Alto, CA, USA) vectors for functional testing. All the plasmids included a copy of the enhanced fluorescent protein (EYFP) [13].

### 2.3. Cancer Cells Transfected with Polyethyleneimine and Antioxidant Treatment

Large-scale transfection was performed using polyethyleneimine (PEI; Sigma Aldrich, 408727) [30]. First, 5 × 10^5^ A549 cells were seeded on 6-well culture plates in preparation for the transfection. Then, the following day, both PEI (3.2 μg) and recombinant plasmid (3.2 μg) were mixed, and the transfection was executed. In addition, the group treated with N-acetyl-L-cysteine (NAC, 2 mM) (Sigma, Catalog No. A7250) was used in the PEI (3.2 μg) and the recombinant plasmid (3.2 μg) group for 48 hpt.

### 2.4. Separation of Mitochondria from B2-Gene-Transfected Cancer Cells

To detect the morphological changes occurring in the mitochondria, A549 cells (NSCLC) were seeded in 60 mm diameter culture dishes at a cell density of 10^5^ cells/mL in 4 mL of the aforementioned medium for a total of 24 h. The cells were subsequently transfected with EYFP-B2, EYFP-∆B2, or EYFP. For a total of 48 h post transfection, the B2-transfected cells were stained with a mitochondria signal staining reagent (MitoTracker tRed CM-H2XRos, Invitrogen) according to the included protocol [29]. Then, the B2-transfected cells were imaged using a standard fluorescence microscope and induced at 488 and 510 nm with a 515 and 590 nm long-pass filter, respectively, for fluorescence excitation [29].

Mitochondrial isolation was achieved by modifying a reported protocol. Briefly, 2 × 10^6^ cells/mL were washed with PBS and homogenized with the help of a glass homogenizer in 0.3 mL of mitochondria isolation buffer (10 mM HEPES, 0.1% bovine serum albumin, and 0.35 M mannitol; pH 7.2). Subsequently, the cells were centrifuged (600× *g* for 10 min at 4 °C) to obtain a pellet and centrifuged further (10,000× *g* for 15 min at 4 °C) to isolate a mitochondrial pellet. The supernatant was then retrieved and combined with 20 μL of 10× concentrated SDS buffer, which was boiled and run through a Western blotting procedure [8].

### 2.5. In Vitro Detection of Relative Hydrogen Peroxide Levels by H2DCFDA 

ROS generation in the B2-transfected A549 cells was calculated using a fluorescent cytometry assay based on H2DCFDA intracellular oxidation (Life Technologies, Carlsbad, CA, USA) [12]. Briefly, the cells were cultured in a 6-well plate overnight, after which their media were replaced with B2 transfection media; then, cells they were incubated for 48 h. The samples were washed further with PBS in preparation of staining, which lasted 30 min at 37 °C. Green fluorescence was imaged using a standard fluorescence microscope, with excitation values at 488 and 515 nm.

### 2.6. A549 Human Lung Cancer Cell Xenograft Model in NOD/SCID Mice

Transfected A549 tumor cell lines (1 × 10^6^/mouse in 100 μL 0.9 % saline) were injected subcutaneously into the flank of a male NOD/SCID mouse [31] to induce solid tumors. About 15 days following the inoculation, test drugs (5-Fu, 0.9% in saline: *n* = 5) and agents (PEI/vehicle: *n* = 4; PEI/flag: *n* = 6; PEI/flag-B2: *n* = 5; and PEI/flag-ΔB2: *n* = 6) were mixed for 30 min and left to stand for 1–2 h, followed by administration via intratumoral injection (thrice per week; 12 times in total) for 4 weeks (Table 1). The tumor size and mouse weight were measured once per week. All the mice (*n* = 4–6/group) were sacrificed on day 35. Tumors were collected and processed for H&E staining (*n* = 4), and their sera were used for blood biochemistry analysis.

### 2.7. Immunostaining with Antibodies for Tumor Markers

Sections of the tumors and organs in the animals with and without the B2 treatment were processed for immunohistochemistry staining. First, paraffin-embedded slides were treated with xylene and a series of different doses of ethanol. Antibodies against Napsin (Cell Signaling Technology), CD133 (Cell Signaling Technology), and Thy1 (D3V8A) (Cell Signaling Technology) were used. The IHC staining signal was semiquantitatively analyzed using the ImageJ program. The data (All *n* = 3) are representative of three separate experiments, and the error bars represent the SEMs; * *p* < 0.05 and ** *p* < 0.01.

### 2.8. Analysis of qRT-PCR for Tumor Tissues

Total RNA was extracted from different solid tissues with the help of TRIzol (Invitrogen Corp., Carlsbad, CA, USA) as indicated by the manufacturer’s protocol. To prepare cDNA pools for each sample of RNA, total RNA (5 μg) was reverse-transcribed with the help of a TOOLS Easy Fast RT kit. The mixture was incubated at 42 °C for 15 min, followed by incubation at 95 °C for 3 min. The cDNA concentration was determined with a NanoDrop ND-1000 spectrophotometer. For each qPCR analysis, approximately 200 ng of cDNA was used [12]. QPCR with oligonucleotide primers specific to the constitutively expressed gene β-actin was used to normalize all the samples and ensure the fidelity of the mRNA extraction and reverse transcription procedures. Table 2 presents the primer sequences obtained in the study. All qPCRs were performed using the TOOLS 2X SYBR qPCR Mix kit. The cycling conditions of the thermal cycler (LightCycler^®^ Nano) were as follows: an initial denaturation cycle at 95 °C for 10 min followed by 40 cycles of 95 °C for 15 s, 60 °C for 15 s, and 72 °C for 20 s [12,32].

### 2.9. Western Blot Analysis

Following the harvesting procedure, whole-cell extracts were prepared with 3% BSA, 1× PBS, and 0.1% Tween-20 and subsequently lysed with 0.05% SDS. The samples were then boiled for 2 min and centrifuged (10,000× *g* at 4 °C for 10 min) to obtain the supernatant. Then, dilutions with 6× Laemmli loading buffer were performed on the supernatant, followed by a further 2 min of boiling prior to loading. Proteins were run under 10% sodium dodecyl sulfate–polyacrylamide gel electrophoresis (SDS-PAGE) [8]. The resulting membranes were blocked in blocking solution (0.1% Tween-20, 3% BSA, and 1× TBS) for at least 1 h at room temperature. Primary antibodies were used to immunoblot the samples overnight at 4 °C as follows: anti-RGNNV B2 polyclonal antibodies (self-prepared), anti-FLAG monoclonal antibodies (Cell Signaling; CAT: No.2968), anti-Nrf2 (Enzo; CAT: ADI- KAP-TF125-F), anti-p38 (Cell Signaling; CAT: No.8690), anti-phosph-p38 (Cell Signaling; CAT: No.4511), anti-voltage-dependent anion channel (VDAC) (Santra-Cruz; CAT: sc-390996) MAb, anti-Nrf2 (ENZO, Code No. Q16236) MAb, anti-catalase (Rockland, Code No. 200-401-051) MAb, anti-MnSOD (GeneTex, GTX 116093) MAb, Cu/ZnSOD (Cayman Chemical, Code No. 10011388) MAb, P53 (Cell Signaling; CAT:9282) Mab, BAX (Cell Signaling; CAT:2772) Mab, RIP3 (Proteintech; CAT:17563-1-AP) Mab, and anti-β-actin (Calbiochen; CAT: No. MAB1501) MAb. The membranes were then washed with TBST (with 0.1% Tween-20 and TBS) and incubated with 1:2000 diluted secondary antibody (horseradish peroxidase, DakoCytomation) at room temperature for 1 h. After being washed further, the membranes were analyzed with an enhanced chemiluminescence system (ECL, Amersham Life Sciences), and ImageJ computer software was used to quantify the signals. For the loading controls, β-actin was used [8].

### 2.10. Statistical Analysis

GraphPad Prism 8.2.1 and SPSS 16.0 software were used for all statistical analyses. We used Student’s *t*-test to assess the significance of differences between the two groups and one-way analysis of variance (ANOVA) followed by Tukey’s post hoc test to assess the significance of differences between multiple groups. Where relevant, bars in the graphs are presented as the mean ± SD or mean ± SEM for at least three experimental replicates. Statistical significance is shown at * *p* < 0.05 and ** *p* < 0.01 [13].

## 3. Results

### 3.1. Use of a Novel Signaling Peptide in Mitochondrial Targeting

**Figure 1:**
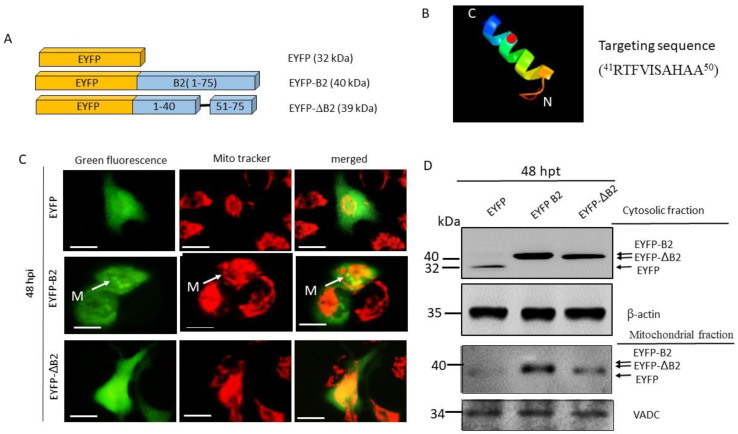
Identification of a novel mitochondria-targeting sequence from B2 in lung cancer cells. (**A**) Mitochondrion-targeting sequences designed using different constructs of wild-type and point mutant forms of RGNNV B2 protein. (**B**) Three-dimensional alpha–helix structure of the full-length RGNNV B2 (1–75 aa) and its mitochondrion-targeting region (36–61 aa). N: N terminus; C: C terminus. (**C**) Trancing EYFP-B2 fusion protein targeted in mitochondria by fluorescence spectrometric analysis 48 h post transfection produces yellow fluorescence in 45% of A549 cells (see Figure S1) but not in cells with EYFP and EYFP-ΔB2 (del^41^RTFVISAHAA^50)^). Green, fluorescent, Mitotracker staining and merged images of EYFP, EYFP-B2, and EYFP-ΔB2 transfected cancer cells 48 h post transfection, with the EYFP-B2 fusion protein targeted in mitochondria shown in yellow and indicated by arrowheads (white bar: 10 μm). M: mitochondria. (**D**) Western blot analysis of EYFP shows the protein distribution in the mitochondrial and cytosolic fractions at 48 h post transfection.

Non-structural protein B2 obtained in betanodavirus subgenome RNA 3 has recently been presented to target mitochondria using a novel signaling peptide (^41^RTFVISAHAA^50^) [12,13]. We set out to determine whether B2 can also target mitochondria in human A549 in a similar manner. Mutant EYFP-ΔB2 with the targeting region deleted and functional EYFP-B2 were also used (Figure 1A). Our analysis predicted B2 to have an alpha–helix structure (Figure 1B) [12]. MitoTracker Green was used to examine the localization of the B2 protein. Our results indicate yellow fluorescence in the whole mitochondria of lung cancer cells that express EYFP-B2 (Figure 1C). In contrast, the cells expressing EYFP and the EYFP-ΔB2 group (Figure 1A) present with fluorescence almost exclusively in the cytoplasm. Subsequently, we analyzed B2 colocalization with mitochondria using Western blotting 48 h post transfection (pt) (Figure 1D). The results reveal that B2 can target mitochondria in all A549 cells expressing full-length EYFP-B2 and in very few or no cells in the other groups, suggesting that the specific signal peptide is used to target mitochondria in A549 cancer cells.

### 3.2. Triggering of Hydrogen Peroxide/Nrf2-Mediated Stress Signals by B2 Targeting In Vitro

**Figure 2:**
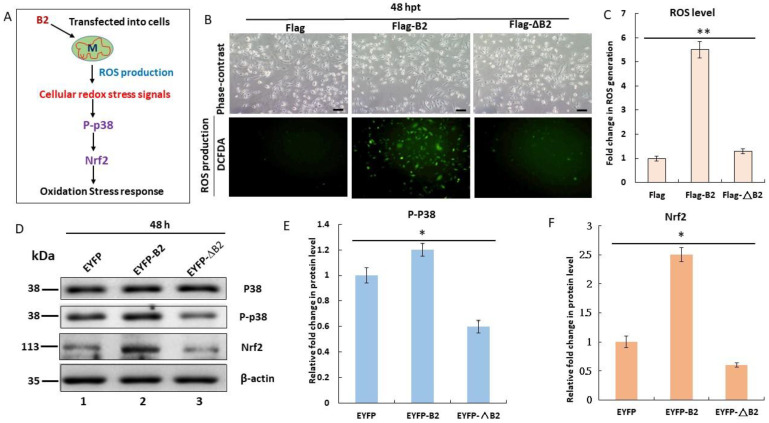
B2 protein causes an ROS/Nrf2-mediated redox oxidative stress signal following mitochondrial targeting in A549 cells. (**A**) Schematic representation of B2-induced, ROS/Nrf2-mediated stress response in A549 cells 48 h post transfection. (**B**) Identification of B2-induced ROS production in different A549 cells transfected with Flag, Flag-B2, and Flag-ΔB2 plasmids. ROS production was traced using the DCFDA method. (**C**) Quantification of ROS production. The data are representative of three separate experiments, and the bars represent the SEMs. * *p* < 0.05 and ** *p* < 0.01, as analyzed by one-way ANOVA with Tukey’s multiple comparisons. (**D**) Identification of p38 phosphorylation sites and Nrf2 expression in A549 cells by Western blot analysis. (**E**,**F**) Quantification of the results in D. The data are representative of three separate experiments, and the error bars represent the SEMs. * *p* < 0.05 and ** *p* < 0.01, as analyzed by one-way ANOVA with Tukey’s multiple comparisons.

Previous studies conducted on fish have demonstrated that mitochondrial targeting by B2 is linked with cellular ROS production [29]. However, the exact mechanism underlying ROS-mediated signaling remains unknown. Therefore, we examined ROS-mediated signaling in A549 cells (Figure 2A). Using an H_2_DCFDA assay, we observed that cells transfected with B2 had four times higher ROS production 48 h pt compared to the hydrogen-peroxide-production-positive control, Flag, and Flag-ΔB2 groups (Figure 2B,C). Then, Western blotting (Figure 2D) revealed that the phosphorylation of downstream molecule p38 increased by 0.2-fold in Flag and by 0.6-fold in Flag-ΔB2 (Figure 2E) and that the expression of Nrf2 (Figure 2F) was reduced by 1.5-fold in EYFP and by 1.9-fold in EYFP-ΔB2. These results reveal that B2-activated mitochondria can trigger p38/Nrf2-mediated stress signals in the early expression stage of A549 lung cancer cells.

### 3.3. Blockage of B2-Mediated Nrf2 Stress Signals by Antioxidant NAC, Reducing Antioxidant Enzyme Expression in A549 Cells

**Figure 3:**
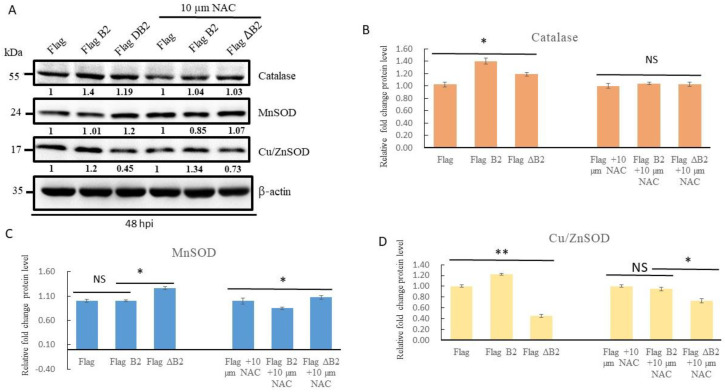
Identification of NAC-inhibited B2 protein-induced stress markers in A549 cells. (**A**) Suppression of B2 (2 mM)-mitochondria-targeting-induced catalase, MnSOD, and Cu/ZnSOD expression in A549 cells by Western blot analysis. The group treated with N-acetyl-L-cysteine (NAC, 2 mM) was used in the PEI (3.2 μg) and the recombinant plasmid (3.2 μg) group for 48 hpt. (**B**–**D**) Quantification of the results in A. The data are representative of three separate experiments, and the bars represent the SEMs. * *p* < 0.05 and ** *p* < 0.01, NS = not significant, as analyzed by one-way ANOVA with Tukey’s multiple comparisons.

To test the function of B2-mediated hydrogen peroxide/Nrf2-mediated stress signals, we treated the antioxidant NAC and observed its effect on an A549 lung cancer cell with B2 transfection and expression. At 48 h pt (Figure 3A), we observed that the antioxidant NAC was able to suppress the hydrogen peroxide/Nrf2-mediated stress signals to inhibit oxidative stress by monitoring the antioxidant enzymes, catalase, MnSOD, and Cu/ZnSOD expression levels by Western blot analysis. The B2 protein induced catalase expression by up to 0.4-fold compared to Flag (as a normal control; 1-fold) and 0.2-fold compared to Flag-ΔB2 (0.21-fold) (Figure 3B). On the other hand, the NAC-treated group suppressed catalase upregulation compared to the Flag and Flag-ΔB2 groups. For MnSOD expression (Figure 3C), B2 protein did not induce catalase expression (1.01-fold); such expression was induced instead by the Flag-ΔB2 group (1.2-fold) compared to the Flag group (1-fold). In the NAC-treated group, we observed that the Flag-B2 group presented with lower expression than the Flag and ΔB2 groups. Then, for Cu/ZnSOD expression (Figure 3C), we observed that the B2 protein induced increased expression by up to 0.2-fold in the Flag group and 0.75-fold in the Flag-ΔB2 group. In the NAC-treated group, the expression in the Flag-B2 group (0.95-fold) was less than that in the Flag group (1-fold); however, this did not reduce the expression values in the Flag-ΔB2 group (0.73-fold) (Figure 3D).

### 3.4. Reducing Solid Tumors in NOD/SCID Mice by B2 Expression

**Figure 4:**
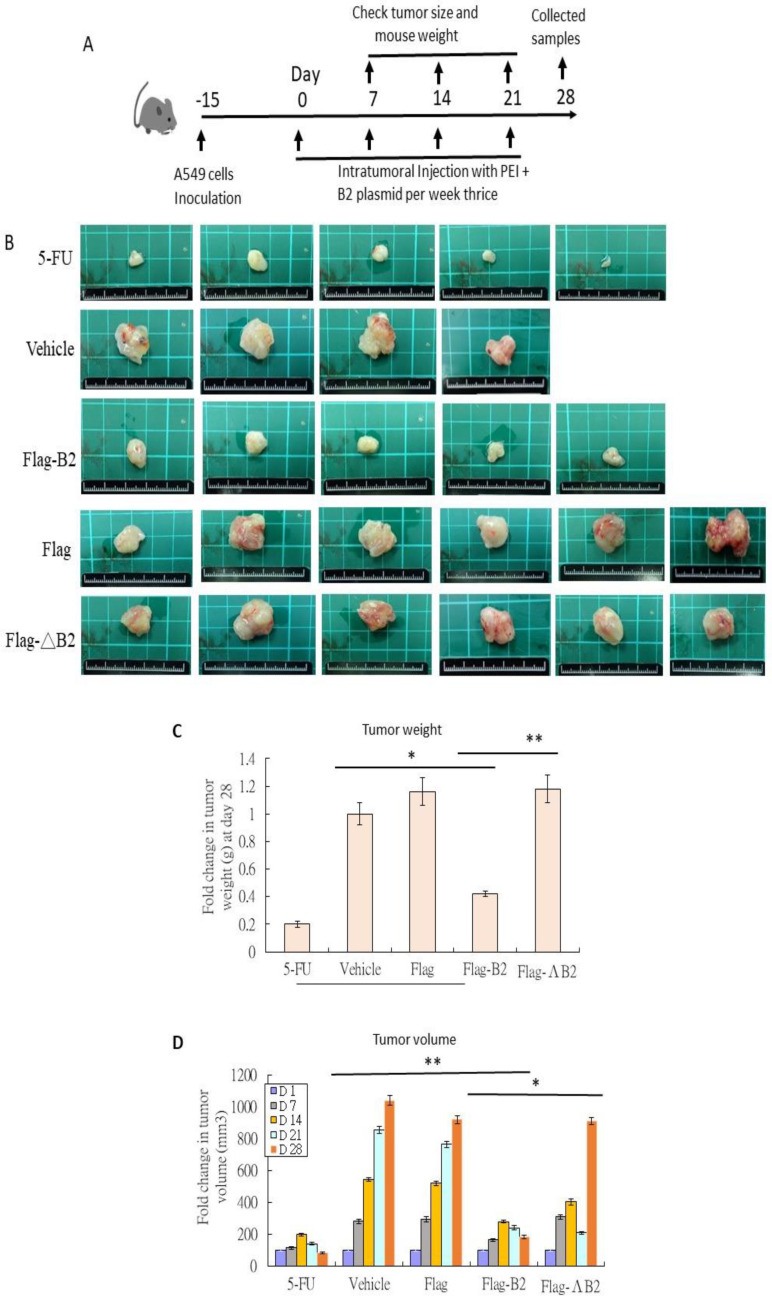
B2 protein can kill A549 lung cancer cells in an NOD/SCID mouse. (**A,B**) Tumor samples suggesting that *B2* gene injections block cancer growth 28 days after 5-FU (positive control; *N* = 5), vehicle (negative control; *N* = 4), Flag-B2 (*N* = 5), Flag (*N* = 6), and Flag-ΔB2 (mutant control) treatments (*N* = 6; see Table 1). Quantitative analysis of (**C**) tumor weight and (**D**) tumor volume of samples presented in Figure 3B. *N* = 4–6. The data are representative of three separate experiments, and the error bars represent the SEMs. The data were analyzed by one-way ANOVA with Tukey’s multiple comparison test, with *p*-values defined as * *p* < 0.05 and ** *p* < 0.01 compared to the control groups.

To test the function of B2 protein, we examined its effect on an A549 lung cancer cell-induced solid tumor grafted in NOD/SCID mice (Figure 4A; Table 1). After 28 days, we observed that the B2 protein killed A549 cancer cell-induced solid tumors more efficiently (Figure 4B; *N* = 4–6) than the 5-FU (commercial, positive control), vehicle, Flag, and Flag-ΔB2 (mutant control) groups. B2 also reduced the tumor weight (Figure 4C) by 5.8-, 7.4-, and 7.6-fold compared to the vehicle, Flag, and Flag-ΔB2 groups, respectively. Furthermore, we monitored the tumor volume on days 1, 7, 14, 21, and 28 and observed that in the Flag-B2 group, the tumor volume was dramatically reduced by 5.6-, 5.0-, and 5.0-fold compared to the vehicle, Flag, and Flag-ΔB2 groups, respectively (Figure 4D).

### 3.5. Reducing B2-Triggering Stress Signals in Solid Tumors in NOD/SCID Mice

**Figure 5:**
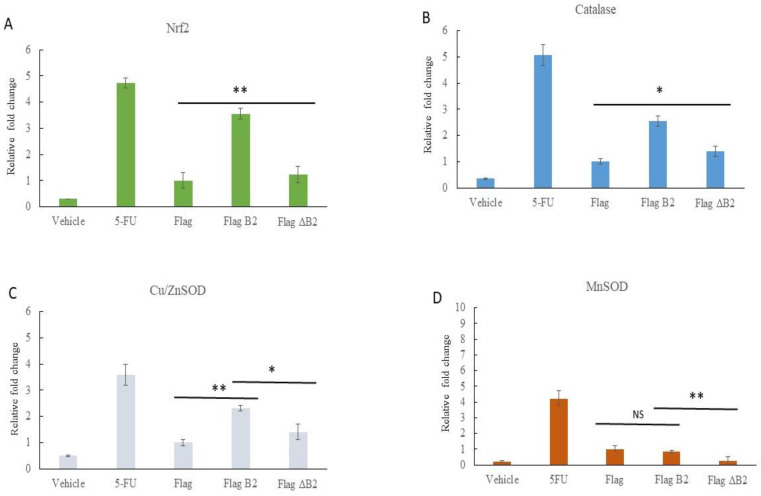

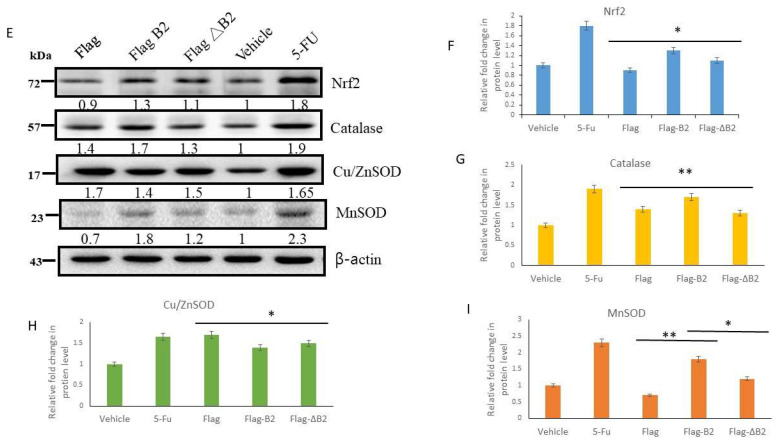
B2 protein induces ROS/Nrf2-mediated redox oxidative stress signals via stress markers catalase, Cu/ZnSOD, and MnSOD in NOD/SCID mice. RT-qPCR analysis of oxidative stress signal via stress markers (**A**) Nrf2, (**B**) catalase, (**C**) Cu/ZnSOD, and (**D**) MnSOD, suggesting the induction of a ROS/Nrf2-mediated redox oxidative stress signal in solid tumors (*N* = 4–6). The data are representative of three separate experiments, and the error bars represent the SEMs. * *p* < 0.05 and ** *p* < 0.01, as analyzed by one-way ANOVA with Tukey’s multiple comparisons. (**E**) Upregulation of B2-mitochondria-targeting-induced catalase, MnSOD, and Cu/ZnSOD expression in A549 cell solid tumors according to Western blot analysis. (**F**–**I**) Quantification of the results in E. The data are representative of three separate experiments, and the bars represent the SEMs. * *p* < 0.05 and ** *p* < 0.01, NS = not significant, as analyzed by one-way ANOVA with Tukey’s multiple comparisons.

In order to test the marker genes of B2-protein-induced stress signals, such as Nrf2, catalase, MnSOD, and Cu/ZnSOD, at the mRNA level, we examined its effect on an A549 lung cancer cell-induced solid tumor grafted on NOD/SCID mice (Figure 4B and Figure 5) using the real-time q-PCR approach. After 28 days, in the solid tumors, we observed that the B2 protein efficiently induced oxidative stress in the A549 cancer cell-induced solid tumor (Figure 5), vehicle, Flag, and Flag-ΔB2 (mutant control) groups. B2 also induced increased Nrf2, catalase, and Cu/ZnSOD expression by up to 2.3- (Figure 5A), 1.1- (Figure 5B), and 0.9-fold (Figure 5C) compared to the vehicle, Flag, and Flag-ΔB2 groups, respectively, but did not induce MnSOD expression (Figure 5D). The 5-FU (commercial positive control) also presented a strong induction of oxidative stress response in the antioxidant enzymes presented in Figure 5A–D. Furthermore, in our Western blot analysis of protein expression levels, we found that the B2 protein efficiently induced oxidative stress in the A549 cancer cell-induced solid tumor (Figure 5E), vehicle, Flag, and Flag-ΔB2 (mutant control) groups. B2 also induced increased Nrf2, catalase, and MnSOD expression by up to 0.2- (Figure 5F), 0.5- (Figure 5G), and 0.9-fold (Figure 5I) compared to the vehicle, Flag, and Flag-ΔB2 groups, respectively, but did not induce Cu/ZnSOD expression (Figure 5H). The 5-FU (commercial positive control) also presented a strong induction oxidative stress response in the antioxidant enzymes presented in Figure 5E–I. Taken together, the data on oxidative stress marker genes reveal that Nrf2 and catalase are correlated with the mRNA and the protein expression level. On the other hand, Cu/ZnSOD and MnSOD have minor differences between in terms of their mRNA and protein levels, but the overall trends are similar in response to oxidative stress.

### 3.6. Triggering Two Death Types in p53/Bax and the RIPK3-Mediated Cell Death Pathway

**Figure 6:**
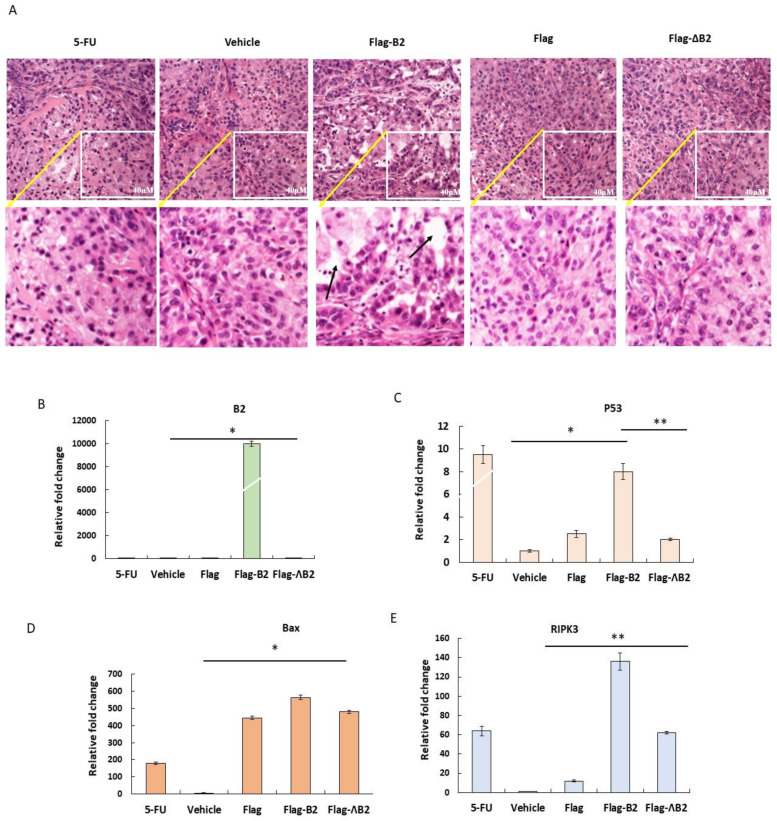

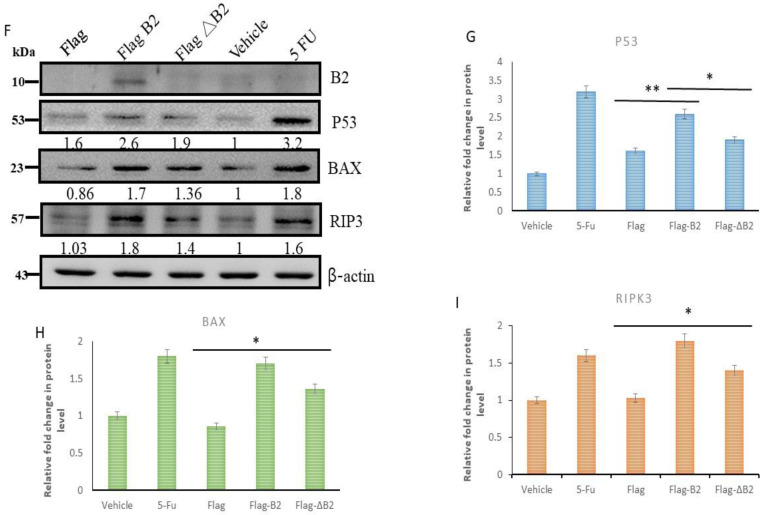
B2 expression can trigger two different cell death pathways. (**A**) Tissue section analysis of A549 cell solid tumors by H&E staining. Cell damage sites are indicated by arrows in B2-expressing cells. RT-qPCR analysis of gene expression of (**B**) B2 and mRNA expression of (**C**) p53 and (**D**) Bax, suggesting the induction of the p53/Bax-mediated apoptotic pathway. (**E**) RT-qPCR analysis of RIPK3, suggesting the induction of RIPK3-mediated necroptosis in solid tumors (*N* = 4–6). The data are representative of three separate experiments, and the error bars represent the SEMs. * *p* < 0.05 and ** *p* < 0.01, as analyzed by one-way ANOVA with Tukey’s multiple comparisons. (**F**) Upregulation of the B2-mitochondria-targeting-induced P53/Bax-mediated apoptosis death pathway (**G**,**H**) and the RIPK3-mediated necroptosis pathway (**I**) in A549 cell solid tumors according to Western blot analysis. (**F**–**I**) Quantification of the results in F. The data are representative of three separate experiments, and the bars represent the SEMs. * *p* < 0.05 and ** *p* < 0.01, as analyzed by one-way ANOVA with Tukey’s multiple comparisons.

In our previous study, we showed that the B2 protein can kill A549 and H1299 cell lines. Therefore, we analyzed tumor tissues obtained from different groups using H&E staining to determine the cell death ratio on day 28 (Figure 6A). We observed a void in the cells treated with Flag-B2 in place of A529 cancer cells (indicated by the arrows) when compared to the H&E staining of cells treated with the vehicle, Flag, and Flag-ΔB2. To verify that this void was caused by B2, we probed the B2 gene expression using RT-qPCR in all groups and observed that B2 gene expression presented a 1000-fold change in the Flag-B2 group (Figure 6B) compared to the other groups. We then analyzed the cell death signaling pathways and determined an upregulation of *p53* and *Bax* genes in the Flag-B2 group (Figure 6C,D) compared to the other groups. Furthermore, we observed that the RIPK3-mediated necroptosis signal was triggered in the B2 expression group but not in the other groups (Figure 6E). Furthermore, to verify that this void was caused by B2, we probed the B2 protein expression level using Western blot analysis in all the groups and observed that B2 protein expression presented a fivefold change in the Flag-B2 group (Figure 6F) compared to the other groups. We then analyzed the cell death signaling pathways and determined an expression of p53 and Bax proteins in the Flag-B2 group (Figure 6G,H) compared to the other groups. Furthermore, we observed that the RIPK3-mediated necroptosis signal was triggered in the B2 expression group (Figure 6I), which was consistent with results of mRNA and protein expression level. On the other hand, the 5-FU (commercial positive control) also presented a strong induction of apoptosis and necroptosis responses in the P53, Bax, and RIPK3, as presented in Figure 6F–I, showing a minor difference between the mRNA and protein level with respect to Bax and RIPK3.

### 3.7. In Vivo Inhibition of Cancer Marker Expression In Vivo B2 Protein

**Figure 7:**
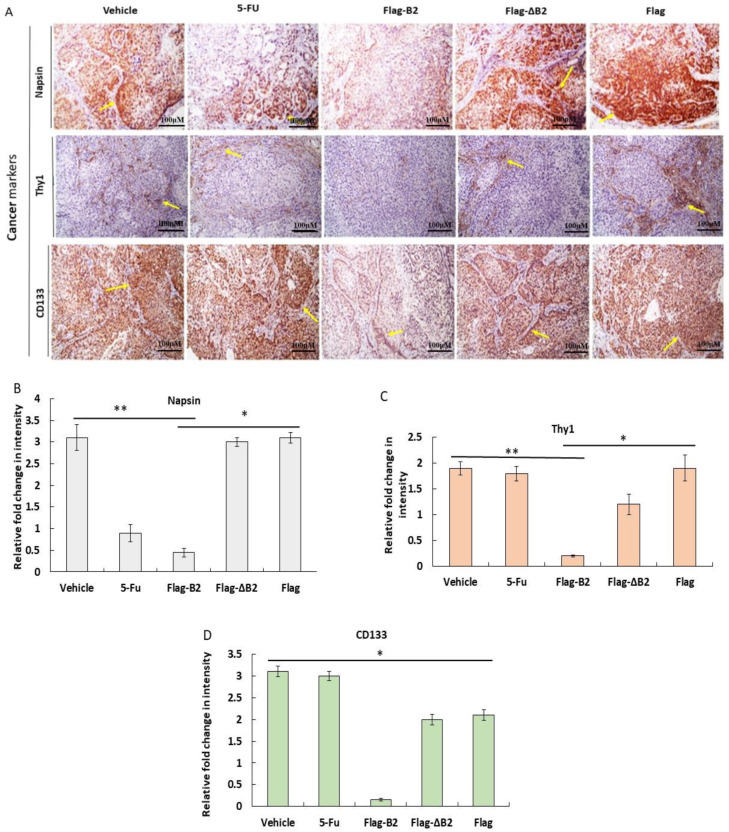
B2-mediated oxidative stress in solid tumors can reduce the expression of tumor markers napsin, Thy1, and CD133 in NOD/SCID mice. (**A**) Solid tumors obtained from the lungs of NOD/SCID mice that underwent treatment with 5-FU (positive control), vehicle (negative control), Flag-B2, Flag, and Flag-ΔB2 (mutant control) were immunostained with anti-napsin, anti-Thy1, and anti-CD133 antibodies. Scale bar = 100 uM. Quantification of signal intensity of (**B**) napsin, (**C**) Thy1, and (**D**) CD133 (All *N* = 3). The data are representative of three separate experiments, and the error bars represent the SEMs. * *p* < 0.05 and ** *p* < 0.01, as analyzed by one-way ANOVA with Tukey’s multiple comparisons.

Cancer markers are important for cancer cell invasion and migration during clinical metastasis. Thus, we aimed to trace cancer markers such as CD133 [33,34], Thy1 [35,36], and napsin [37,38] in solid tumor tissues using immunostaining and counted them using an ImageJ programmed system (N = 4) (Figure 7A). In the Flag-B2 group, CD133, Thy1, and napsin were present at lower levels than in the vehicle, Flag, and Flag-ΔB2 groups (Figure 7B). On the other hand, 5-FU treatment did not repress CD133, Thy1, or napsin expression. Therefore, we concluded that B2 can suppress the expression of cancer markers in A549 cancer cells.

In summary, our results show that the B2 protein uses a novel signal peptide to target mitochondria, which correlates to the triggering of ROS/p38/Nrf2 oxidative stress in A549 cancer cells. Then, the B2-triggered signal cascades into a mixed-type cell death induction through the P53/Bax- and RIPK3-involved pathways. These pathways lead to the expression inhibition of tumor markers related to tumor migration. Thus, B2 is a potential therapeutic protein drug target for the treatment of lung cancer.

## 4. Discussion

The novel B2 protein induces necroptosis and the malfunction of mitochondria in aquatic fish cells, resulting in ROS induction and ATP loss from mitochondria. Furthermore, B2 can induce cell death in different cancer cell lines, such as epithelial cervical cancer, breast adenocarcinoma, and lung adenocarcinoma cells [8,13], which also activate apoptosis signaling. These results suggest that the B2 protein can play a novel role in killing A549 lung cancer cells in NOD/SCID mice [39] via the ROS/Nrf2-mediated triggering of multiple pathways that control cell death and cell migration.

### 4.1. B2 Induces Mitochondrion-Mediated Hydrogen Peroxide/Nrf2 Signals in Lung Cancer Cells

Reactive oxygen species (ROS) are reactive and unstable compounds formed from incompletely reduced oxygen derivatives as a byproduct of normal metabolism. ROS include compounds, such as singlet oxygen (^1^O_2_), superoxide anion (O_2_^−^), hydrogen peroxide (H_2_O_2_), hypochlorous acid (HOCl), and hydroxyl radicals (·OH). They also play the role of secondary messengers, regulating downstream gene transcripts required for numerous biological functions in both healthy and cancerous cells [39,40]. Balanced intracellular ROS levels are maintained by antioxidant enzymes, such as glutathione (GSH), catalase (CAT), and thioredoxin (Txn), which metabolize ROS species [13] and maintain homeostasis [41]. The production of ROS is a strategy observed in most chemotherapies due to their involvement in triggering cell death signaling. Thus, ROS are a type of tumor suppressant [42]. Recently, some evidence has suggested that prolonged chemotherapy can reduce total ROS values within tumors [43]. In our study, we observed that the B2 protein has a presequence segment that typically consists of 15–40 amino acid residues rich in hydroxylated (mostly serine) and positively charged residues (Figure 1) [44,45,46]. Furthermore, B2 expression induced hydrogen peroxide (H_2_O_2_) production [13,47] (Figure 2B) and acted as a signaling molecule that triggered p38/Nrf2 stress signals, which correlated with oxidative-stress-marker enzymes, such as catalase, Cu/ZnSOD, and MnSOD, for upregulation both in vitro (Figure 3) and in vivo (Figure 5). These signals were strongly correlated with reduced cancer growth and controlling characteristics—an effect not observed in the ΔB2 mutant control (Figure 4). Furthermore, this oxidative stress signal could be interrupted to reduce the stress response.

### 4.2. Why Can B2 Trigger Multiple Signals for Death Control In Vivo?

P53 is crucial for cellular survival and is regarded as the protectant of the genome [48]. Nearly all cancers present multiple function changes in p53 [49,50,51], revealing its significance as a tumor suppressor. Furthermore, p53 upregulates some genes in different manipulating functions, including ROS metabolism, apoptosis, senescence, and cell cycle arrest [41,47,49,52,53,54,55]. That being said, the signals that affect a cell’s fate following p53 expression remain poorly understood in the literature [56].

In our system, A549 (p53^+/+^) human lung cancer cells induced a solid tumor in NOD/SCID mice that was eliminated upon B2 expression. We observed that the targeting of mitochondria by B2 triggered ROS/Nrf2-mediated stress signals and induced cell death signaling pathways via p53/Bax-mediated apoptotic and RIPK3-mediated necroptotic signaling (Figure 6E). However, mutant ΔB2 did not induce these signals. Tumor microenvironments are often divided into distinct classifications based on their histology and prevailing interactions with non-cancerous cells [57,58,59]. Thus, these group classifications can either be spatially different (such as hypoxic and perivascular regions) or separated according to their main cellular interactions (such as the immune niche), creating a diverse and dynamic tumor ecology [60]. Accordingly, our results lead us to propose a possible novel mechanism that may present a new perspective on cancer treatment [61,62,63,64,65].

### 4.3. Can a B2-Triggering ROS/Nrf-2 Stress Response Regulate Stem Cell Marker Expression In Vivo?

Recently, the Nrf2–Keap1 system has been perceived to be a fundamental component of the cellular response that controls a large variety of transcriptional targets that are mainly involved in the regulation of redox homeostasis and multiple cytoprotective mechanisms that confer adaptation to stress conditions [66,67,68]. Subsequently, the pleiotropic response orchestrated by Nrf2 is particularly relevant in the context of oncogenic activation, where this transcription factor acts as a key driver of tumor progression and cancer cell resistance to treatment. Additionally, the influence of Nrf2 on cancer cell biology extends far beyond its mere antioxidant function and encompasses a functional crosstalk with the mitochondrial network. Interestingly, Nrf2 can influence crucial aspects of mitochondrial homeostasis, including biogenesis, oxidative phosphorylation, metabolic reprogramming, and mitophagy. Moreover, Nrf2 can engage in crosstalk with mitochondria, with a particular focus on malignant tumors and cancer stem cells [69].

In our study, we observed that B2 targeted mitochondria and induced an ROS/Nrf2-mediated stress response that triggered p53/Bax-mediated apoptosis and RIPK3-mediated necroptosis. This was achieved by suppressing cancer cell markers CD133, Thy1, and napsin (Figure 7), as presented in NOD/SCID mice. The latter experimental model provided a more complex microenvironment than the in vitro experiments. This activity correlated with the inhibition of cancer migration and metastasis, suggesting a novel role in cancer cell regulation. In our system, B2 derived from a viral gene triggered an ROS/Nrf2-mediated stress response, which activated multiple signals, eventually leading to host cell death induction; however, this stress signal of cancer cell marker induction is not well-known in the literature and still requires additional focus and testing.

**Figure 8:**
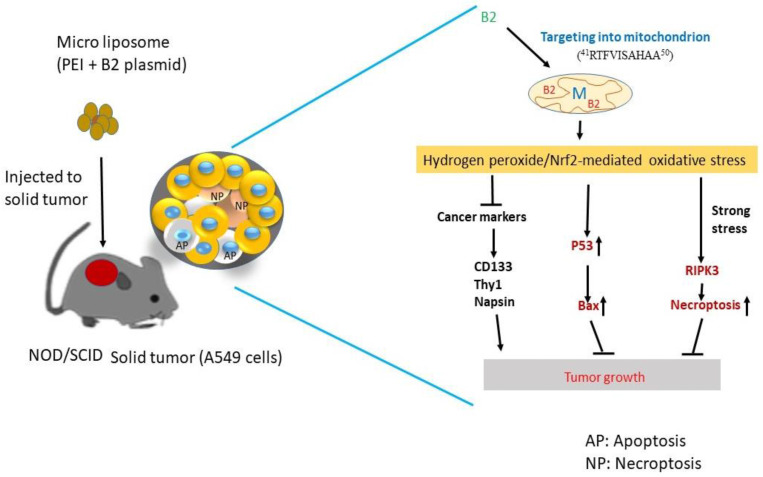
Hypothesized effect of mitochondrion-targeting protein B2 on multiple signaling pathways by ROS-mediated oxidative stress in cancer cells. The expression of the B2 gene in A549 lung tumor cells or in A549 cell-induced solid tumors in NOD/SCID mice induced ROS-mediated stress signals, causing p53/Bax-mediated apoptosis and RIPK3-mediated necroptosis. The B2 protein can transfer into the mitochondria using its novel transferring signal sequence. ^41^RTFVISAHAA^50^. It then regulates complex II activity, suppressing ATP production and enhancing ROS generation, thereby repressing cancer cell growth. B2 triggers ROS/Nrf2-mediated stress, further affecting multiple signaling pathways, such as (1) the induction of the p53- and Bax-mediated apoptotic signals and the RIPK3-mediated necroptosis signal, as well as (2) a reduction in the cancer marker expressions of CD133, Thy1, and napsin in mice, which are responsible for cell migration and invasion in lung tumors.

In summary (Figure 8), the mitochondrion-targeting B2 protein induced ROS/p38/Nrf2-mediated oxidative stress and triggered multiple signals, resulting in the induction of both the p53/Bax-apoptotic pathway and the RIPK3-mediated necroptotic pathway to kill A549 lung cancer cells and suppressed tumor marker expression, such as that of CD133, Thy1, and napsin, which regulate cancer cell migration and metastasis. This discovery may provide a novel perspective on lung cancer management and protein therapy.

## Figures and Tables

**Table 1:** Materials and drugs used in experiment.

Group		No	Agents	Dose	Characteristics of Test Drugs
A	A549	5	5-FU	20 mg/kg body weight	
B	A549	4	0.9% saline	50 µL	
C	A549	6	PEI/Flag	25 µg/25 µg in 50 µL	Agents (PEI, Flag, Flag b2, or Flag ΔB2) are mixed for 30 min at 40 °C and will be stable for 1–2 h at 4 °C
D	A549	5	PEI/Flag b2	25 µg/25 µg in 50 µL	
E	A549	6	PEI/Flag ΔB2	25 µg/25 µg in 50 µL	

**Table 2:** Primer used in the experiment.

Name	Sequence (5′-3′)
p53 Forward primer	AGGGTTAGTTTACAATCAGC
p53 Reverse primer	GGTAGGTGCAAATGCC
Bax Forward primer	GGTGCCTCAGGATGCG
Bax Reverse primer	GGAGTCTGTGTCCACG
Actin Forward primer	ATCCGCAAAGACCTGT
Actin Reverse primer	GGGTGTAACGCAACTAAG
RGNNV B2 Forward primer	ATGGCAAATCCAACAAGC
RGNNV B2 Reverse primer	CTAGTCCGTCTCCATCGGCT
Ripk3 Forward primer	GACTCCCGGCTTAGAAGGACT
Ripk3 Reverse primer	CTGCTCTTGAGCTGAGACAGG
Catalase Forward primer	AACTGGGATCTTGTGGGAA
Catalase Reverse primer	GACAGTTCACAGGTATCTG
Cu/Zn Forward primer	GCGACGAAGGCCGTGTGCGTTG
Cu/Zn Reverse primer	TGTGCGGCCAATGATGCAATG
Mn Forward primer	CGACCTGCCCTACGACTACGG
Mn Reverse primer	CAAGCCAACCCCAACCTGAGC
Nrf2 Forward primer	ACACGGTCCACAGCTCATC
Nrf2 Reverse primer	TGTCAATCAAATCCATGTCCTG

## Data Availability

The data are contained within this article.

## Supplementary Materials

The following supporting information can be downloaded at: https://www.mdpi.com/article/10.3390/biomedicines11010186/s1, Figure S1: Established EYFP protein expression condition in A549 lung cancer cells at different time points.
