# Supplementary material for: A Mitochondrion-Targeting Protein (B2) Primes ROS/Nrf2-Mediated Stress Signals, Triggering Apoptosis and Necroptosis in Lung Cancer
Source: Biomedicines. 2023 Jan 11;11(1):186. doi: 10.3390/biomedicines11010186 (PMC9855812; doi:10.3390/biomedicines11010186)
Supplement: Supplementary file 1 [file biomedicines-11-00186-s001.zip › biomedicines-1981016-supplementary.pdf]

Figure S1

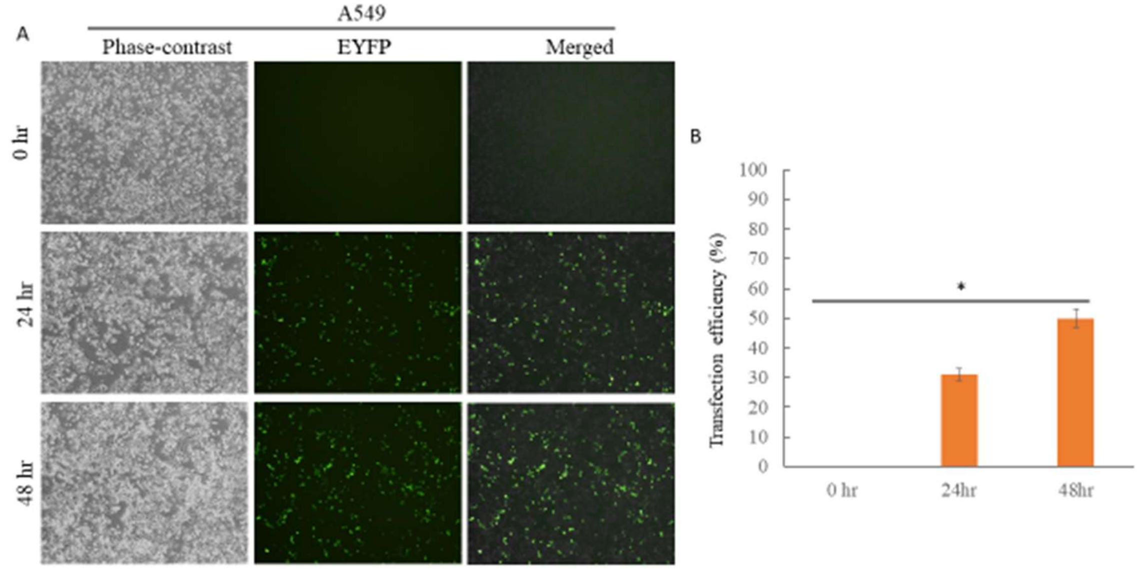


**Figure S1. Established EYFP protein expression condition in A549 lung cancer cells at different time points**. (A) Identification of plasmid of EYFP transfected with Lipofectamine 2000 for 0 h, 24 h, and 48 h post-transfection. At the end of the time points, fluorescence spectrometry analysis at 0 h, 24 h, and 48 hpt showed yellow fluorescence in A549 cells. (B) Quantification of the results in A. The data are representative of three separate experiments, and the bars represent the SEMs. **P* < 0.05, analyzed by one-way ANOVA with Tukey's multiple comparisons.
